# 

**Published:** 1871-10

**Authors:** 


PUBLICATIONS, ETC.—The various publications received will be acknowledged and noticed in the next number of the JOURNAL.

ALL communications and exchanges should be addressed to the ATLANTA MEDICAL AND SURGICAL JOURNAL, care of "The Plantation Publishing Company," Atlanta, Georgia.

